# Effect of fish meal substitution with trout viscera protein hydrolysate on the innate immune response of red tilapia (*Oreochromis* spp)

**DOI:** 10.1007/s10695-024-01444-0

**Published:** 2025-02-27

**Authors:** Wilmer Sanguino-Ortiz, Cristóbal Espinosa-Ruiz, María Ángeles Esteban Abad, Críspulo Perea Román, José Luis Hoyos-Concha

**Affiliations:** 1 Department of Hydrobiological Resources, Faculty of Livestock Sciences, Torobajo University Citadel, University of Nariño, 52001 Nariño, Colombia; 2 Immunobiology for Aquaculture Group, Department of Cell Biology and Histology, Faculty of Biology, Regional Campus of International Excellence “Campus Mare Nostrum”, University of Murcia, 30100 Murcia, Spain; 3 Faculty of Agricultural Sciences, University of Cauca, Vereda Las Guacas, 190003 Cauca, Colombia

**Keywords:** Aquaculture, Bactericidal activity, Fish hydrolysate, Lysozyme activity, Mucosal immunity, Red tilapia

## Abstract

This study evaluated the effect of replacing fish meal (FM) with concentrated trout viscera protein hydrolysate (TVPH) on the immune response in juvenile red tilapia (*Oreocheromis* spp). Five isoenergetic and isoproteic experimental diets were prepared by substituting FM with TVPH at different substitution ratios: 0% (control, D1) 25% (D2), 50% (D3), 75% (D4), and 100% (D5). A total of 180 red tilapia were distributed in 15 tanks. Fish from three tanks were fed daily at 2% of the biomass for 25 days with one of the five diets mentioned above. At the end of the trial, the fish were counted, weighed, measured, and skin mucus and serum samples were obtained to study different parameters related to humoral immunity. The results indicate a 100% survival rate in all fish groups and did not show significant differences in terms of growth and feed efficiency. On the other hand, the fish fed diets D2 and D3 had significantly higher serum protein values. Also, fish fed the D2 or D5 diets had higher lysozyme activity and fish fed the D2 diet also had significantly higher total immunoglobulin levels than fish fed the control diet. In mucus, fish fed the D2 or D4 diets showed significantly higher mucus protein levels than control fish. However, anti-protease and bactericidal activity decreased in fish fed the D5 or D4 diets, respectively. These results demonstrate that the D2 and D3 diets positively modulate the immune response of juvenile red tilapia compared to that of fish fed the control diet.

**Supplementary Information:**

The online version contains supplementary material available at 10.1007/s10695-024-01444-0.

## Introduction

In 2020, it was estimated that approximately 29.5% of the 178 million tonnes of fish generated by fisheries and aquaculture went towards fishmeal and fish oil production (FAO 2022; FAO et al. 2020). Additionally, a portion of the fish waste, ranging from 20 to 80% of the total volume, was used for this purpose, while the rest was discarded into the environment (FAO 2022; FAO et al. 2020; Gao et al. 2021). It is anticipated that fish consumption will increase by 15% by 2030, resulting in an increase in fish waste (Das et al. 2021; Estiasih et al. 2021; FAO 2022) However, fish waste is a significant source of nutrients and energy that is equal to fishmeal and fish oil used in animal feed manufacturing (FAO 2022; Hoyos-Concha et al. 2018).

One of the challenges is that fishmeal currently does not meet the demand for livestock production, leading to a significant increase in its price and compromising its quality and availability (Díaz-Cachay et al. 2023; Hoyos-Concha et al. 2018; Perea Román et al. 2021). As a result, aquaculture production, which involves raising fish at high densities, requires the consumption of high-cost artificial feeds (Dediu et al. 2021; Montoya-Mejía 2016; Vásquez et al. 2013; Wurmann G 2019). These feeds must contain adequate levels of protein, energy, fatty acids, essential amino acids, and phospholipids, among other nutrients necessary for optimal production processes (Behera et al. 2018; Briones García 2021; Kitiyodom et al. 2021).

However, inadequate feed supply in fish subjected to high stocking and compromised water quality facilitates the proliferation of pathogens that can eventually lead to disease and severe mortalities (Behera et al. 2018; Briones García 2021). Furthermore, stress caused by overcrowding and/or poor nutrition affects growth, survival, and immunity (both cellular and humoral) (Brum et al. 2017; Laith et al. 2019; Suprayudi et al. 2017). More specifically, stress alters overall homeostasis and causes stressed animals to suffer more diseases, morbidities, and mortalities, which also results in fish suffering from diseases, causing substantial economic losses (Abdel-Ghany and Salem 2020; Hoyos-Concha et al. 2022a, b). From the above, it can be deduced that it is important to have balanced fish diets that have quality raw materials and that provide all the nutritional requirements of production fish to ensure their proper growth, welfare, and health. In addition, there is an urgent need to find partial or total substitutes for fishmeal (Abdel-Ghany and Salem 2020; Arteaga et al. 2022; Román et al. 2017).

Owing to their nutritional and functional properties, hydrolyzed fish protein products are of great interest in the food, pharmaceutical, and cosmetic industries, among others (Goosen et al. 2016; Perea Román et al. 2021; Yathisha et al. 2022). In addition, these hydrolysates have antioxidant, antihypertensive, antithrombotic, antibacterial, and antiobesogenic properties, which make them important for aquaculture (Daroit and Brandelli 2021; Kaur et al. 2021; Osman et al. 2021). In addition, the use of fish protein hydrolysates as an additive to fishmeal reduces feed intake, improves digestion, absorption, and the functioning of the immune system of animals (Gao et al. 2021), and improves immune response and disease resistance in juvenile barramundi (*Lates calcarifer*) (Siddik et al. 2018). One of the sources used to obtain hydrolyzed protein is the viscera of rainbow trout (*Oncorhynchus mykiss*), which in 2020 reached a production of 739.5 thousand tonnes (FAO 2022). The total trout waste is between 50 and 70%, depending of transformation processes used (Vásquez et al. 2022). In addition, viscera constitute 16% of the live weight and are sources of proteins, digestive enzymes, lipids, and bioactive compounds (Villamil et al. 2017). Hoyos-Concha and Gaviria-Acosta (2021) patented hydrolyzed protein meal for animal feed processes, obtained from trout viscera, and found that it had antioxidant and antimicrobial capacity. In addition, its high digestibility, adequate nutritional profile and essential amino acids make it an ideal raw material to partially replace fishmeal, as both raw materials are similar in their proximate composition and amino acid profile (Hoyos-Concha et al. 2018, 2022a, b). Likewise, the product has a pH of less than 4.0 and is free of total coliforms, aerobic mesophiles, molds, and yeasts (Perea Román et al. 2021).

In view of the above, this study evaluated the effect of replacing fishmeal (FM) with concentrated protein hydrolysate obtained from trout viscera (TVPH) on the innate immune system of juvenile red tilapia (*Oreochromis* spp*.*) fed experimental diets for 25 days. Subsequently, different activities (total protein and immunoglobulin levels, proteases, antiproteases, peroxidase, lysozyme, and bactericidal activity) were determined in skin mucus and serum, and the antioxidant capacity in serum was determined. The choice of red tilapia species is based on its global importance and production volume of 1.3 million tonnes in 2022, which is one of the most consumed species, positioning it within the main group of aquaculture products in intensive and super-intensive systems (FAO 2022; Ministerio de Agricultura y Desarrollo Rural 2021).

## Materials and methods

### Trout viscera protein hydrolysate (TVPH) and experimental diets

Forty kilograms of rainbow trout (*Oncorhynchus mykiss*) viscera were used as raw material, obtained from local fish farms in Cauca (Colombia). This material was processed in the bioassay and biotechnology laboratories of the University of Cauca, Colombia, in an 80-L plastic tank at room temperature (Hoyos-Concha and Gaviria-Acosta 2021). Briefly, 2.5% of 85% formic acid, 0.25% sodium benzoate, and 0.1% butylated hydroxytoluene were manually mixed for 10 min to facilitate the activation of endogenous enzymes and initiate the hydrolysis process, which lasted 6 days. The process was stopped by a water bath, raising the temperature to 50 °C for 30 min to denature the endogenous enzymes, and then the hydrolysate was allowed to cool. Afterwards, it was dried at 70 °C for 16 h, then it was centrifuged (243 × *g*, 30 min) and the precipitate was passed through a centrifugal separator (340 × *g*, 70 Hz, 40 min) to remove most of the water and oil, to obtain concentrated hydrolyzed protein.

Five isoenergetic and isoproteic experimental diets were formulated for red tilapia, in which FM was replaced by TVPH in the following proportions: 100% FM control (D1), 75% FM and 25% TVPH (D2), 50% FM and 50% TVPH (D3), 25% FM and 75% TVPH (D4), and 100% TVPH (D5) (Hoyos-Concha et al. 2018; Román et al. 2018, 2021) (Supplement Table 1). The raw materials were ground, sieved (< 425 µm), and weighed, and micronutrients and macronutrients were added to them. Everything was mixed in a mixer (SIMAG SM-401) for 20 min at 25 °C. Then, the resulting mixes were passed through an industrial twin-screw extruder (Hake Polylab) at 123 °C to obtain pellets with a diameter of 4.5 mm and a length of 5 mm with a maximum moisture content of 10%. The diets were allowed to cool and were packed in hermetically sealed bags for later use (Román et al. 2017).

The proximate and energetic analysis of the experimental diets was carried out in the specialized laboratories of the University of Nariño (Colombia), following the Official Methods of Analysis (AOAC) as indicated by other authors (Costa et al. 2020; Latimer 2023; Mendes et al. 2024; Siddaiah et al. 2022). Briefly, dry matter was obtained by drying using the gravimetric technique at 105 °C for 24 h (AOAC 934.01), ash content was obtained by muffle ashing with gravimetry at 405 °C for 24 h (AOAC 942.05), crude protein was measured as total nitrogen (N) using the Kjeldahl method, with N multiplied by a factor 6.25 (AOAC 968.06) ethereal extract by Soxhlet extraction with gravimetric analysis at 150 °C (AOAC 920.39), calcium wet oxidation with spectroscopy (AOAC 968.08), phosphorus wet oxidation with photometric analysis (AOAC 965.17), and gross energy with bomb calorimetry (Román et al. 2021).

### Fish, experimental design, and sampling

The experiment was approved by the Ethics Committee for Scientific Research of the University of Cauca, Colombia (act N° 6.1–1.25 of August 15, 2019). A total of 180 red tilapia (*Oreochromis* spp.) from a local fish farm (Cauca, Colombia) were adapted to laboratory conditions and fed a commercial diet ad libitum for 20 days. At the end of the adaptation period, with an initial average weight of 110 ± 10 g and a mean total length of 18 ± 0.5 cm, the fish were randomly distributed into 15 tanks (250 L) equipped with aeration, heating, and freshwater recirculation systems. Water quality parameters were monitored weekly during the adaptation and experimental periods using a photometer (YSI 9500) and an oximeter (YSI Pro 20). Temperature was between 27 and 28 °C, dissolved oxygen between 5 and 6 mg L^−1^, pH between 6.8 and 7.2, and the concentration of nitrogen compounds was < 0.01 mg L^−1^. Fish in each of the tanks (in triplicate) were fed 2% of their biomass (distributed into two meals per day) with one of the experimental diets for 25 days, according to previous studies (Goosen et al. 2016; Mahdhi et al. 2020; Perea Román et al. 2021).

Fish were sedated with 20 mg L^−1^ clove oil (MON) after a 24-h fast to proceed to sample collection (Albaladejo-Riad et al. 2023). Skin mucus samples were obtained with the aid of a silicone spatula by superficially removing the mucus laterally in an anterior–posterior direction on both sides of the fish, avoiding contamination with other body fluids (Guardiola et al. 2014). Samples were centrifuged (10,062 × *g*, 4 °C, 10 min) and supernatants were collected and frozen at − 80 °C until further use. Blood samples were obtained from the gill chamber by puncturing the aortic arch using insulin syringes without anticoagulant. Samples were placed in test tubes and refrigerated at 4 °C for 4 h. Serum was collected from each sample, centrifuged (10,062 × *g*, 4 °C, 10 min), placed in Eppendorfs and frozen at − 80 °C until use (Guardiola et al. 2014). The immune parameter analyses in serum and skin mucus of fish were conducted in the laboratories of the Faculty of Biology at the University of Murcia, Spain.

### Growth performance

At the end of the experiment, all the fish of each group were, weighed, measured, and counted to obtain survival rates (S%), weight gain (WG%), feed conversion ratio (FCR), and specific growth rate (SGR, % day⁻^1^), according to the following equations (Messina et al. 2023; Perea Román et al. 2021):

$$\begin{array}{l}WG\%=\left[\left(final\;weight-initial\;weight\right)/initial\;weight\right]\times100\\SGR\%\;{day}^{-1}=\left[\text{ln}\left(final\;weight\right)-ln\left(initial\;weihgt\right)\times100\right]/days\end{array}$$

### Total protein

Serum and mucus protein values were measured in triplicate according to the Bradford method, using bovine serum albumin (BSA, Sigma-Aldrich) as standard (Espinosa-Ruiz et al. 2021). For this, 5 µL of the mucus sample was placed in each well of the flat-bottomed plate (Nunc) containing 5 µL of the standard and 250 µL of Bradford was added. The samples were left in the dark with shaking. The absorbance of each sample was read at 590 nm and the results were calculated according to the standard curve to obtain the total protein content of serum and mucus.

### Immune parameters in serum and skin mucus

Peroxidase activity was measured by placing 5 µL of serum or mucus sample in triplicate in 96-well flat-bottomed plates. To each well, 45 µL of Hanks’s buffer (HBSS) was added without Ca^+2^ y Mg^+2^ and 100 µL of the solution 3.3′,5.5′-tetramethylbenzidine (TMB, 10 mM) with 0.025% of 30% H_2_O_2,_ when the reaction changed color, the reaction was stopped by adding 50 µL of H_2_SO_4_ 2M and the absorbance was read at 450 nm (Espinosa-Ruiz et al. 2021). Samples without serum and mucus were used as blanks and their absorbance values were subtracted from those obtained in the samples. A unit of peroxidase activity was defined as the amount that produces a change of observance by a value of one and was expressed as U mL^−1^ of serum or mucus protein.

The protease activity was measured only in serum using azocasein hydrolysis (Espinosa-Ruiz et al. 2021). It was measured in Eppendorf tubes in triplicate, and separately, 10 µL of serum and 100 µL of mucus were taken. To serum samples, 100 µL of 0.1 N ammonium bicarbonate was added along with 125 µL of 2% azocasein diluted in ammonium bicarbonate. However, 100 µL of 0.7% azocasein diluted in ammonium bicarbonate was added to mucus samples. All samples were incubated by shaking for 24 h at room temperature. Then, 250 µL of 10% TCA was added to the serum samples. Samples were centrifuged (6000 × *g* × 5 min), and 100 µL was transferred to 96-well flat-bottomed plates and 100 µL of 1N NaOH was added. For the positive control, 100 µL of trypsin was used and for the negative control 100 µL of ammonium bicarbonate was used. Finally, all plates were read in the spectrophotometer at 450 nm.

Anti-protease activity was measured in serum and mucus by determining its ability to inhibit trypsin activity according to the defined methodology (Espinosa-Ruiz et al. 2021). Samples were incubated at room temperature with 10 µL of trypsin for 10 min, then 100 µL of ammonium bicarbonate buffer, 125 µL of azocasein (Sigma) at 0.2% for serum and 0.7% for mucus, both diluted in ammonium bicarbonate respectively, were added, the samples were incubated for 2 h at room temperature. Then, 250 µL of TCA were added at 10% for serum and 4.6% for mucus and incubated for 30 min at 25 °C. Samples were centrifuged (6000 × *g*, 5 min), 100 µL of the supernatant was transferred to 96-well flat-bottomed plates and 100 µL of 1N NaOH for serum and 0.5 N for mucus was added. For positive and negative control, 10 and 20 µL of ammonium bicarbonate were used instead of serum and mucus respectively. Finally, the plate was read in the spectrophotometer at 450 nm, and the results of each sample were expressed as the percentage of inhibited trypsin in relation to the control samples.

Lysozyme activity in serum and mucus samples was determined using 25 µL of each sample diluted 1:2 in 10 mM PBS (pH 6.2) in triplicate in 96-well flat-bottomed plates. To each well, 175 µL of lyophilised *Micrococcus lysodeikticus* diluted in the same buffer (0.3 mg mL^−1^, Sigma) was added as lysozyme substrate. Immediately, each plate was read at 450 nm for 15 min in 3 min cycles at room temperature. A reduction in absorbance of 0.001 min^−1^ was defined as the unit of lysozyme activity min^−1^ (Marcos-López et al. 2017). The resulting lysozyme values in serum and mucus were obtained from the standard curve made with egg white lysozyme (HEWL, Sigma), and the results obtained were expressed as U mg^−1^ of serum or mucus protein.

Immunoglobulin levels in serum and mucus were measured in Eppendorfs in which 10 µL of serum or 50 µL of mucus were placed, then 10 µL and 50 µL of 12% polyethylene glycol, respectively, were added. The samples were shaken and allowed to stand for 2 h at room temperature and centrifuged (5000 × *g*, 10 min). Aliquots of 10 µL of the supernatant of the serum samples were diluted in Eppendorf tubes prepared with 190 µL distilled water, from the mixture and 5 µL was transferred to 96-well flat-bottomed plates. 250 µL of Bradford’s reagent was added to each well and incubated in the dark with shaking at room temperature for 10 min (Albaladejo-Riad et al. 2022). The standard line was defined using bovine serum albumin (BSA) at concentrations of 0, 0.25, 0.5, 0.5, 1, and 1.4. The absorbance of the serum, mucus, and standard samples was read at 590 nm and the level of total immunoglobulins was calculated by the difference of the total protein of each sample and the protein contained in the samples processed with polyethylene glycol.

To determine the bactericidal activity, 20 µL of sample (serum or mucus) was placed in triplicate in 96-well flat-bottomed plates and 20 µL of *Vibrio anguillarum* incubated for 5 h at 25 °C was added to each well. A Hank’s balanced salt solution was used as a positive control. Each well was spiked with 25 µL 3-(4,5-dimethylthiazol-2-yl)−2, 5-diphenyltetrazolium bromide 1 mg mL^−1^, (Sigma-Adrich) to be incubated for 10 min at 25 °C. The plates were centrifuged (2000 × *g*, 10 min). The precipitate was dissolved in 200 µL dimethyl sulfoxide and transferred to 96-well flat-bottomed plates and read at 550 nm (Guebebia et al. 2023). Bactericidal activity was calculated as the difference between the absorbance of surviving bacteria and that of the positive control and the result was expressed as % bactericidal activity.

### Antioxidant activity in serum

The antioxidant capacity of serum was evaluated using the 2.2-azino-bis-3-(ethylbenzothiazoline-6-sulphonic acid) (ABTS) method (Guebebia et al. 2023). In a cuvette, 950 µL of ABTS and 50 µL of serum were added, and the absorbance at 730 nm was measured immediately at 0, 30, and 60 s to determine the amount of ABTS radical cations reduced by the antioxidants in the sample, as per the ascorbic acid standard curve. The amount of ABTS consumed by the sample was calculated using its molar extinction coefficient (ε) of 13,000 M^−1^ cm^−1^, considering that 1 mol of ascorbic acid reduces two moles of ABTS.

### Statistical analysis

The data presented in the figures are expressed as the mean ± standard deviation (SD). The statistical analysis was performed using the SPSS for Windows® software (version 25.0, SPSS Inc., Chicago, IL, USA). Prior to analysis, we conducted a test to determine the homogeneity of variances using Levene’s test and assessed the normality of the variables using the Shapiro–Wilk test. An unrestricted randomized experimental design was employed, and significant differences between the five experimental groups (diets) were determined using one-way analysis of variance (ANOVA) with a *p*-value > 0.05. Post hoc tests, including Tukey’s test or Games-Howell test at a 95% confidence level, were applied as necessary to account for significant differences in the variables.

## Results

### Growth performances

The growth and performance results of red tilapia juveniles in terms of WG%, FCR, and SGR, after being fed the experimental diets, indicate that there were no significant differences (*P* > 0.05) between the fish groups fed the experimental diets with FM substituted by TVPH and the fish group fed the control diet (*P* > 0.05) for any of these parameters (Table 1). The survival rate in all fish groups was 100%.

**Table 1:** Growth and productive performance of red tilapia *Oreochromis* spp fed with different levels of trout viscera protein hydrolyzed (TVPH) over 25 days

Parameters	D1 (control)	D2	D3	D4	D5	*P* value
WG %	18.06 ± 3.62	24.78 ± 3.43	21.88 ± 3.38	20.08 ± 2.86	15.20 ± 10.97	0.37
FCR	1.83 ± 0.33	1.47 ± 0.15	1.61 ± 0.24	1.71 ± 0.19	1.9 ± 0.48	0.46
SGR	0.66 ± 0.13	0.89 ± 0.11	0.79 ± 0.11	0.73 ± 0.10	0.56 ± 0.39	0.38

Values are presented as mean ± standard deviation (SD) (*n* = 3)

*WG%* weight gain percentage, *FCR* feed conversion ratio, *SGR* specific growth rate, *D* diet

### Serum and skin mucus parameters

**Fig. 1:**
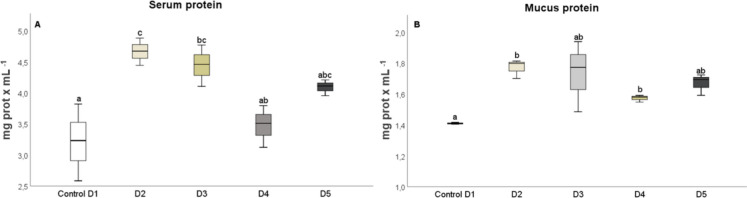
Protein content determined in serum (**A**) and skin mucus (**B**) of red tilapia fed different diets (D1: control, D2, D3, D4, and D5) with varying percentages of protein substitution using trout viscera protein hydrolysate (0%, 50%, 75%, and 100% substitution, respectively). Results are expressed in milligrams of protein per milliliter (mg prot × mL⁻^1^). Bars represent the mean ± standard deviation (SD) (*n* = 3), along with quartiles. Data were statistically analyzed using one-way ANOVA followed by post hoc Tukey’s test (*P* < 0.05), significant differences are indicated by different letters

**Fig. 2:**
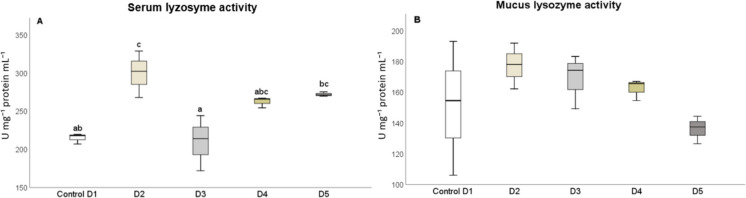
Lysozyme activity determined in serum (**A**) and skin mucus (**B**) of red tilapia fed different diets (D1: control, D2, D3, D4, and D5) with varying percentages of protein substitution using trout viscera protein hydrolysate (0%, 50%, 75%, and 100% substitution, respectively). Results are expressed in units of milligrams per milliliter of protein (U mg⁻^1^ protein mL⁻^1^). Bars represent the mean ± standard deviation (SD) (*n* = 3), along with quartiles. Data were statistically analyzed using one-way ANOVA followed by post hoc Tukey’s test (*P* < 0.05), significant differences are indicated by different letters

**Fig. 3:**
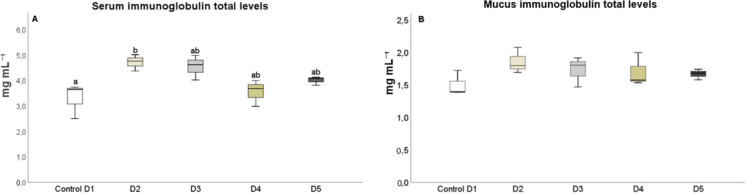
Total immunoglobulin content determined in serum (**A**) and skin mucus (**B**) of red tilapia fed different diets (D1: control, D2, D3, D4, and D5) with varying percentages of protein substitution using trout viscera protein hydrolysate (0%, 50%, 75%, and 100% substitution, respectively). Results are expressed in milligrams of immunoglobulins per milliliter (mg mL⁻^1^). Bars represent the mean ± standard deviation (SD) (*n* = 3), along with quartiles. Data were statistically analyzed using one-way ANOVA followed by post hoc Tukey’s test (*P* < 0.05), significant differences are indicated by different letters

**Fig. 4:**
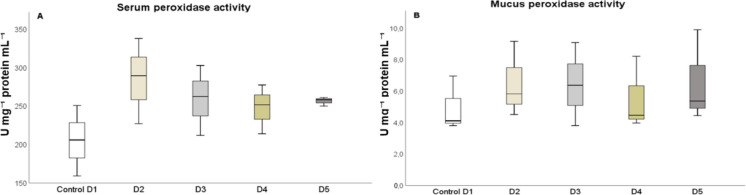
Peroxidase activity determined in serum (**A**) and skin mucus (**B**) of red tilapia fed different diets (D1: control, D2, D3, D4, and D5) with varying percentages of protein substitution using trout viscera protein hydrolysate (0%, 50%, 75%, and 100% substitution, respectively). Results are expressed in units of milligrams per milliliter of protein (U mg⁻^1^ protein mL⁻^1^). Bars represent the mean ± standard deviation (SD) (*n* = 3), along with quartiles. Data were statistically analyzed using one-way ANOVA

**Fig. 5:**
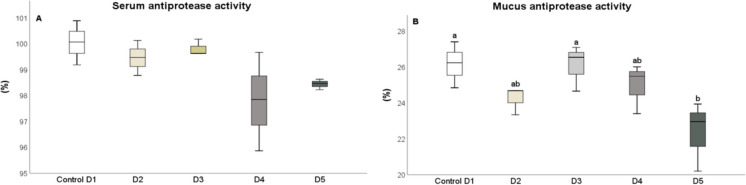
Antiprotease activity determined in serum (**A**) and skin mucus (**B**) of red tilapia fed different diets (D1: control, D2, D3, D4, and D5) with varying percentages of protein substitution using trout viscera protein hydrolysate (0%, 50%, 75%, and 100% substitution, respectively). Results are expressed as a percentage (%). Bars represent the mean ± standard deviation (SD) (*n* = 3), along with quartiles. Data were statistically analyzed using one-way ANOVA followed by post hoc Tukey’s test (*P* < 0.05), significant differences are indicated by different letters

After 25 days, the levels of humoral immune parameters in both the serum and skin mucus were measured in fish that were fed the experimental diets. The total protein content in the serum of fish fed diets D2 and D3 (Fig. 1A) and in the skin mucus of fish fed D2 and D4 (Fig. 1B), respectively, significantly increased (*P* < 0.05) compared to the control diet. The results indicated that the suero lysozyme activity significantly increased (*P* < 0.05) in fish fed diets D2 and D5, relative to the control and D3 diets (Fig. 2A). However, there was no significant difference in lysozyme activity in the skin mucus of fish fed the different diets compared to the control group (Fig. 2B). Although total immunoglobulin levels in the serum increased significantly (*P* < 0.05) in fish fed diet D2 relative to the control diet group (Fig. 3A), there was no significant difference in the levels of total immunoglobulins in the skin mucus of fish fed the experimental diets relative to the levels in fish fed the control diet (Fig. 3B). Neither peroxidase (Fig. 4) nor antiprotease activity (Fig. 5A) in the serum or skin mucus was significantly affected by the ingestion of the experimental diets. However, in the skin mucus of fish fed diet D5, activity significantly decreased (*P* < 0.05) compared to the control group (Fig. 5B). Serum protease activity was not detected in any of the groups of fish fed the experimental diets.

### Bactericidal activity

**Fig. 6:**
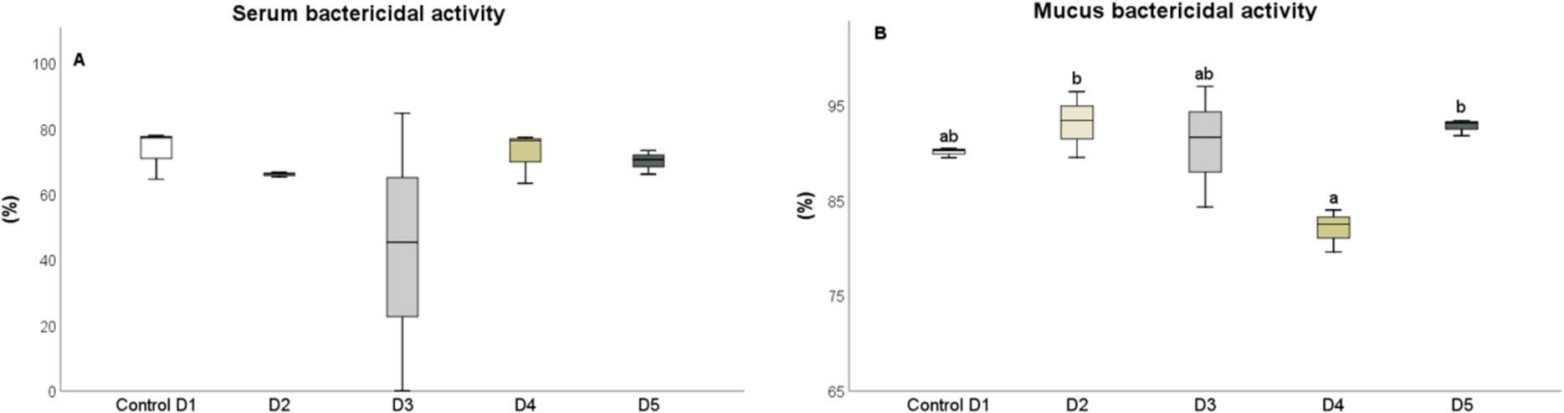
Bactericidal activity determined in serum (**A**) and skin mucus (**B**) of red tilapia fed different diets (D1: control, D2, D3, D4, and D5) with varying percentages of protein substitution using Trout Viscera Protein Hydrolysate (0%, 50%, 75%, and 100% substitution, respectively). Results are expressed as a percentage (%). Bars represent the mean ± standard deviation (SD) (*n* = 3), along with quartiles. Data were statistically analyzed using one-way ANOVA followed by post hoc Tukey’s test (*P* < 0.05), significant differences are indicated by different letters

The bactericidal activity in the fish fed the control and experimental diets was not significantly different in the serum, but there was a significant decrease in bactericidal activity in the skin mucus of fish fed the D4 diet compared to those fed the D2 and D5 diets (Fig. 6B) (*P* < 0.05).

### Serum antioxidant activity

**Fig. 7:**
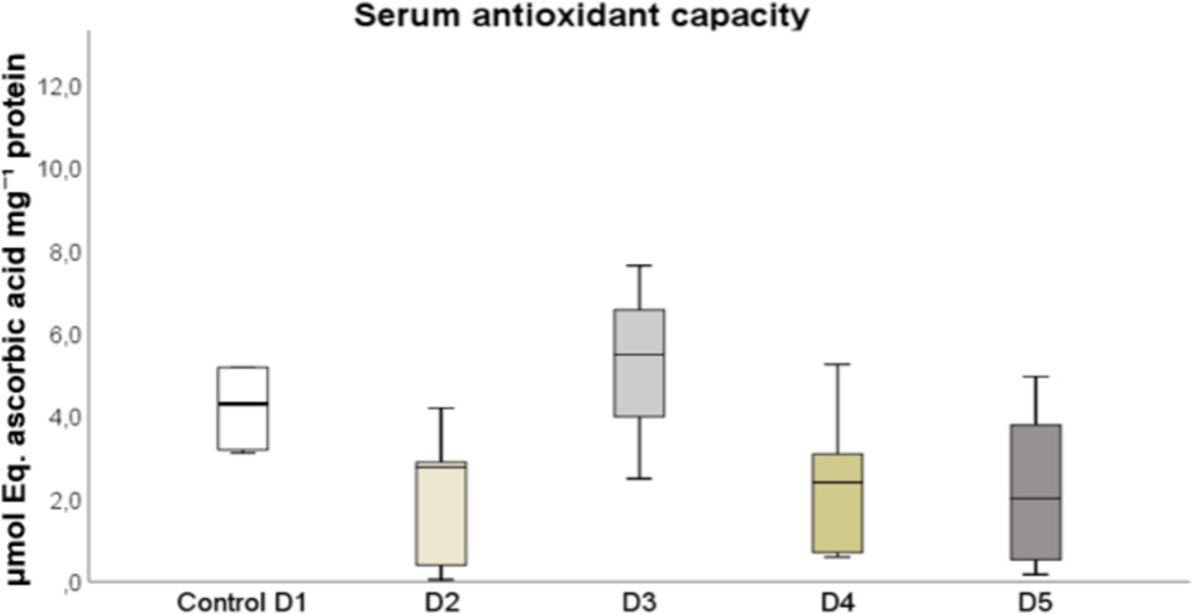
Antioxidant capacity determined in serum of red tilapia fed different diets (D1: control, D2, D3, D4, and D5) with varying percentages of protein substitution using Trout Viscera Protein Hydrolysate (0%, 50%, 75%, and 100% substitution, respectively). Results are expressed as micromolar equivalents of ascorbic acid per milligram of protein (µmol Eq. ascorbic acid mg⁻^1^ protein). Bars represent the mean ± standard deviation (SD) (*n* = 3), along with quartiles. Data were statistically analyzed using one-way ANOVA

There were no significant differences in serum antioxidant activity among the fish fed the different diets (Fig. 7) (*P* > 0.05)*.*

## Discussion

Aquaculture today faces the constant challenge of finding potential raw materials for the substitution of fishmeal (FM) as a main ingredient in feed formulation for feeding fish in production (Nguyen et al. 2023). This research is within this context, evaluating the potential of hydrolyzed trout viscera protein (TVPH) as a substitute for FM. Previous studies highlight the similarity of fish protein hydrolysates (FPH) to FM as well as the benefits found in research in which FPH was fed to captive fish (Hoyos-Concha et al. 2018; Siddaiah et al. 2022). These hydrolysates are characterized by their content of bio-peptides, immunoreactive peptides, antimicrobial peptides, biological activity, and amino acids of high nutritional quality and digestibility that can provide nutritional benefits and improve the non-specific immune response of fish (Daroit and Brandelli 2021; Khieokhajonkhet and Surapon 2020; Nhinh et al. 2023; Vásquez et al. 2022).

The effect of FPH on fish performance is influenced by several factors, including dietary inclusion levels, degree of hydrolysis, size and molecular weight of peptides, amino acid sequence of peptides, dipeptides, tripeptides, hydrolysis methods, and the raw material from which the FPH is obtained (Agustin et al. 2021; Nguyen et al. 2023; Siddaiah et al. 2022). Several previous studies have reported significant benefits in growth and non-specific immune response in fish fed FPH, attributed to the rapid absorption of amino acids and peptides in the gut of fish (Mauricio Rocha et al. 2017; Perea Román et al. 2021; Quinto et al. 2018). Previous studies indicate that fish by-product hydrolysates contain active biopeptides that affect the values of different blood parameters (Daroit and Brandelli 2021). Consequently, the present study represents a novel approach by studying the effects of FM substitution by TVPH obtained by endogenous enzymatic processes on the modulation of the humoral immunity of juvenile red tilapia. In this study, the results showed that all groups of fish fed diets with partial or total replacement of FM with TVPH, compared to fish fed the FM-based control diet, did not affect weight gain, specific growth rate, and feed efficiency, suggesting that replacing FM with TVPH up to 100% did not affect the growth performance of the fish. Previous studies with Mozambique tilapia (*Oreochromis mossambicus*) demonstrated that high levels of fish protein hydrolysate did not result in significant differences in growth, health, or performance (Goosen et al. 2015) The positive results observed in this study can be attributed to the balanced nutritional composition of the diets, the high solubility of TVPH, and the presence of all essential and non-essential amino acids in both quantity and quality. Additionally, the diets were rich in aspartic and glutamic acids, as well as a high concentration of bioavailable peptides with excellent digestibility, making them easily utilized by the fish (Hoyos-Concha et al. 2018; Perea Román et al. 2021).

The growth and survival results of this study are consistent even with 100% FM substitution. This may be attributed to the similarities between TVPH and FM in terms of protein content, energy, amino acid profile, antioxidant capacity, and other factors, which ensure nutrient assimilation comparable to FM (Kabir et al. 2024) These characteristics adequately met the nutritional requirements of the fish. Various studies have confirmed that it is possible to replace FM with fish protein hydrolysates without affecting growth and feed efficiency in juveniles of European sea bass (*Dicentrarchus labrax*), Nile tilapia (*Oreochromis niloticus*) (Kabir et al. 2024; Kotzamanis et al. 2007; Marchi et al. 2024). However, the levels of substitution will depend on the origin of the by-products and the methods of obtaining hydrolysates, as well as the ability of each aquatic organism to assimilate diets with FM replaced by fish protein hydrolysate (Kabir et al. 2024). Other research has reported improvements in the productive performance of striped murrel (*Channa striata*) fed experimental diets with different levels of fish protein hydrolysate inclusion, which is attributed to the short peptide chains and free amino acids produced during the hydrolysis process of the by-product, making the diets more palatable and the peptides more rapidly absorbed by enterocytes than proteins in their natural state (Siddaiah et al. 2022). The survival values found in all fish groups confirm that the replacement of FM with TVPH did not affect this variable, suggesting that the fish welfare regarding their nutritional requirements was maintained, which is consistent with previous studies in Nile tilapia (*Oreochromis niloticus*) reporting values above 92% (Kabir et al. 2024). The red tilapia (*Oreochromis* spp*.*) used in this study, being a hybrid of different tilapia species, may exhibit variations in tolerance and utilization of TVPH compared to other fish species. These differences could result in unique growth and survival outcomes when compared to Mozambique tilapia (*Oreochromis mossambicus*) (Goosen et al. 2015), Nile tilapia (*Oreochromis niloticus*) (Kabir et al. 2024; Kotzamanis et al. 2007; Marchi et al. 2024).

The immune system is a sophisticated network of cells, tissues, and organs designed to protect the body against pathogens and diseases. The innate immune response serves as the body’s initial line of defense, rapidly activating upon pathogen encounter. This response includes physical barriers such as the skin and mucous membranes, as well as innate immune cells. In contrast, the adaptive immune response is a more specialized mechanism that develops over time, providing specific and long-lasting protection (Whyte 2007). Many scientific studies that focus on developing new diets or incorporating functional ingredients tend to prioritize animal growth, often overlooking the potential effects on the immune system. This study aimed to investigate the influence of diet on the innate immune system of tilapia. A beneficial diet would be expected to maintain immune system parameters at stable levels or enhance them. Conversely, a decrease in immune system parameters would indicate a potential negative effect of the diet.

Present results regarding the parameters evaluated in serum, significant increases in total protein levels, lysozyme activity, and total immunoglobulin were observed in fish fed diets containing TVPH. In skin mucus, there was an increase in total protein values and a significant decrease in anti-protease activity and bactericidal capacity. These findings are consistent with previous studies that have documented changes in the non-specific immune response of fish fed diets containing TVPH (Costa et al. 2020; Khosravi et al. 2015; Nguyen et al. 2023; Siddik et al. 2019; Tang et al. 2008).

In this study, diets D2 and D3 with 25% and 50% TVPH substitution increased serum protein values. However, in skin mucus, it was the D2 and D4 diets with 25%–75% substitution that caused this increase. These findings are consistent with results obtained on increased serum protein levels in juvenile *Channa striata* fed diets containing 10% FPH (Siddaiah et al. 2022). Increased protein levels have been reported to be related to the bioavailability of free amino acids, the configuration and molecular weight of peptides and their proportion in FPH that transit and are absorbed in the gut of fish (Costa et al. 2020). In addition, TVPH is known to have a similar amino acid profile to fishmeal, but with a notably higher proportion of peptides with molecular weight between 0.2 and 0.4 KDa (82.4% vs. 20% FM), giving it antimicrobial and antioxidant properties (Hoyos-Concha et al. 2018). The antioxidant properties of the peptides together with the free amino acids present in FPH could contribute to the observed increases in several immune parameters in fish serum (Siddaiah et al. 2022). The results suggest that different levels of protein hydrolysate substitution in the diet have distinct effects on the total protein concentration in serum and mucus. Further studies on the specific proteins present in these matrices are needed to better understand which proteins are increased due to the different substitution levels applied.

Lysozyme activity is a critical indicator of the innate immune response of fish to microorganism attacks, mediating the activation of the complement system and phagocytosis (Gong et al. 2019; Siddaiah et al. 2022). In this work, a significant increase in serum lysozyme activity was observed in fish fed diets containing 25% and 100% TVPH. Previous studies have indicated increases in serum lysozyme activity in different fish species fed low levels of FPH inclusion (Javaherdoust et al. 2020; Siddaiah et al. 2022; Zheng et al. 2013). Interestingly, intermediate levels of TVPH (50% and 75%) did not elicit the same response in serum lysozyme activity as low (25%) or high (100%) inclusion levels. This observation suggests the possibility of a threshold effect, where sufficient peptide availability is achieved only at specific inclusion levels, leading to significant stimulation of lysozyme activity (Tran et al. 2024). Alternatively, a biphasic response could explain these results, where low and high inclusion levels activate immune pathways, while intermediate levels might fail to reach the activation threshold or induce regulatory feedback mechanisms (Oliva-Teles 2012; Suprayudi et al. 2017). Further research is needed to explore the nonlinear relationship between TVPH inclusion and lysozyme activity.

Immunoglobulins play a fundamental role in the immune response of aquatic animals, serving as the primary antibodies involved in defense against antigens through the innate immune system and playing a crucial role in the adaptive immune response of fish (Espinosa-Ruiz et al. 2020; Gong et al. 2019). In this study, a significant increase in total serum immunoglobulins was observed in fish fed diets containing 25% TVPH. Notably, the highest levels of total immunoglobulins were detected in fish consuming the diet with the lowest proportion of TVPH. These findings align with previous studies, which concluded that while appropriate levels of TVPH are beneficial for fish health, excessive inclusion levels may have negative effects (Nhinh et al. 2023).

Bactericidal activity plays an important role within the innate immune system of fish, with the liver being the main organ responsible for the production of proteins with antibacterial properties (Espinosa-Ruiz et al. 2023; Serna-Duque et al. 2023). In this study, a significant decrease in bactericidal activity was observed in skin mucus from fish fed the D4 diet, which contained 75% TVPH. This decrease could be attributed to an increased number of amino acids and peptides flowing into the gut which could have affected the gut and thus the proper absorption of nutrients necessary for the production of antibacterial proteins in the liver of the fish. This is consistent with observed increases in total gut bacterial counts in Nile tilapia (*Oreochromis niloticus*) fed diets containing increased amounts of FPH (Kabir et al. 2024). No similar studies have looked at dietary protein substitution by hydrolysates and studied parameters of immunity in skin mucus, although it is a very important barrier to prevent disease, especially of bacterial origin. Further research is warranted, particularly considering the poorly studied antimicrobial compounds present in such mucus (Díaz-Puertas et al. 2023).

Proteases and antiproteases present in fish play a crucial role in maintaining homeostasis and limiting the ability of bacteria to proliferate and infect the organism (Fatima et al. 2022). Alterations in the balance of proteases and antiproteases may predispose the organism to infections (Albaladejo-Riad et al. 2022). In this study, a significant decrease in antiprotease activity was observed in the skin mucus of fish fed the D5 diet (100% TVPH). These findings align with previous studies that reported negative effects on certain non-specific immune parameters in various fish species fed diets containing high levels of FPH (Goosen et al. 2014; Kabir et al. 2024; Nguyen et al. 2023; Tang et al. 2008).

The observed reduction in antiprotease activity suggests potential negative implications for fish health, including an increased susceptibility to pathogen colonization and infection due to compromised mucosal immunity. This highlights the importance of balancing the inclusion levels of TVPH in the diet to avoid adverse effects on immune defense mechanisms. Future research should focus on identifying optimal inclusion levels of TVPH that enhance growth performance without compromising critical immune parameters, ensuring a balance between nutritional benefits and health maintenance.

This study did not detect significant protease levels in the serum of fish from any diet group. Additionally, measuring protease levels in skin mucus was not possible due to experimental constraints and insufficient mucus collection. Future research is needed to explore the potential effects of TVPH on immune parameters in both serum and skin mucus.

Peroxidase present in the serum and skin mucus plays an important role in the first line of defense of the innate immune system in fish, preventing the colonization of pathogenic microorganisms and maintaining the health of the organism (Espinosa-Ruiz et al. 2020). However, the results of this study indicated no significant changes in serum peroxidase and skin mucus levels in any of the diets containing TVPH compared with the control diet, which contained 100% FM. Similarly, there were no significant changes in bactericidal capacity, antioxidant capacity, antiprotease activity in serum, or in skin mucus for total immunoglobulin levels and lysozyme activity, which were similar to those in fish fed the control diet. Interestingly, as no significant differences in these immune parameters were found in the serum and skin mucus of juvenile red tilapia, it could be suggested that the inclusion of TVPH in the diet did not negatively affect the immune response of the fish and may have even had beneficial effects in some respects. However, it is important to note that other factors such as the specific composition of the diet and TVPH could have influenced the observed results. Previous studies on productive performance and immune response in juvenile sea bass (*Micropterus salmoides*), barramundi (*Lates calcarifer*), and sea bream (*Pagrus major*), fed with different types of FPH at inclusion rates ranging from 10 to 21%, showed improved growth, as well as improvement in innate immune response including lysozyme and antiprotease activity, total immunoglobulin values, as well as disease resistance (Fan et al. 2022; Khosravi et al. 2015; Siddik et al. 2020).

In summary, different levels of hydrolyzed protein substitution (TVPH) in the diet caused significant changes in the tested parameters of the innate immune system of red tilapia. Increases in serum lysozyme activity and total immunoglobulins content, as well as increases in serum and mucus proteins, suggest a possible improvement in the immune response of the fish. These changes, as well as the bactericidal activity in the groups of fish fed the TVPH inclusive diets (25, 50, and 75%) suggest complex interactions between nutrition and the innate immune response in juvenile red tilapia. Further studies on the inclusion of TVPH in fish feed and its effect on the innate immune response could address aspects such as modulation of the gut microbiota, or could focus on trying to optimize feeding practices in aquaculture in order to not only promote optimal growth of fish, but also help to enhance their health and well-being.

## Supplementary Information

Below is the link to the electronic supplementary material.

Supplementary file1 (DOCX 27 kb)

## Data Availability

No datasets were generated or analysed during the current study.
