# Supplementary material for: Effect of fish meal substitution with trout viscera protein hydrolysate on the innate immune response of red tilapia (Oreochromis spp)
Source: Fish Physiol Biochem. 2025 Feb 27;51(2):56. doi: 10.1007/s10695-024-01444-0 (PMC11865115; doi:10.1007/s10695-024-01444-0)
Supplement: Supplementary file 1 — Supplementary file1 (DOCX 27 kb) [file 10695_2024_1444_MOESM1_ESM.docx]

**Suplement Table 1** Formulation and chemical composition of the experimental diets (dry matter basis).

| **Ingredient (g/100 g)** | **D1 (control)** | **D2** | **D3** | **D4** | **D5** |
| --- | --- | --- | --- | --- | --- |
| Fishmeal (FM) | 22.20 | 16.50 | 11.10 | 5.50 | 0.00 |
| Trout viscera protein hydrolyzed (TVPH) ^a^ | 0.00 | 5.50 | 11.10 | 16.50 | 22.20 |
| Soy cake | 14.00 | 14.00 | 14.00 | 14.00 | 14.00 |
| Wheat mogolla | 26.61 | 23.72 | 19.28 | 15.77 | 14.20 |
| Cassava flour | 19.30 | 21.00 | 21.65 | 21.60 | 28.50 |
| Corn bran | 3.00 | 4.00 | 6.50 | 9.40 | 1.00 |
| Yellow corn flour | 0.05 | 0.05 | 0.05 | 0.05 | 1.37 |
| Wheat flour | 0.10 | 0.10 | 0.10 | 0.05 | 0.10 |
| DL-methionine | 0.43 | 0.40 | 0.30 | 0.20 | 0.30 |
| Biomix ^b^ | 2.00 | 2.00 | 2.00 | 2.00 | 2.00 |
| Bicalcium phosphate | 2.13 | 2.61 | 3.58 | 3.81 | 5.00 |
| Calcium carbonate | 0.10 | 0.10 | 0.41 | 1.05 | 0.73 |
| Tryptophan | 0.29 | 0.34 | 0.38 | 0.43 | 0.80 |
| Bentonite | 1.00 | 1.00 | 1.00 | 1.00 | 1.00 |
| Salt | 1.00 | 1.00 | 1.00 | 1.00 | 1.00 |
| Vegetable oil | 7.80 | 7.69 | 7.59 | 7.60 | 7.80 |
| **Chemical composition (g /100 g)** | | | | | |
| Gross energy (Kcal/kg) | 3100.90 | 3100.90 | 3100.90 | 3100.90 | 3100.90 |
| Crude protein (N x 6.25) | 26.18 | 26.18 | 26.18 | 26.18 | 26.18 |
| Crude lipid | 11.00 | 10.89 | 10.92 | 11.10 | 10.44 |
| Ash | 10.37 | 8.93 | 7.57 | 6.17 | 4.69 |

^a^ Trout viscera protein Hydrolyzed (TVPH) was supplied by the University of Cauca (Cauca – Colombia) Chemical composition (% dry matter)— crude protein: 64.52; ether extract: 7.22; ash: 10.42; calcium 2.59. phosphorus; 3.8. Amino acids profile (%): Histidine 3.11; isoleucine 3.83; leucine 6.72; lysine 10.34; methionine 2.47; Phenylalanine 3.44; Threonine 4.57; valine 4.51.

^b^ Composition of vitamin– mineral mixture and addictives – Biomix S.A© (quantity/kg): vitamin A (800,000 UI); vitamin D3 (300,000 UI); vitamin E (11.0 g); vitamin K (2.2 g); vitamin B12 (0.01 g); thiamine (0.6 g); riboflavin (3.6 g); pyridoxine (5.6 g); biotin (0.08 g); pantothenic acid (6.8 g); niacin (5.6 g); folic acid (1.0 g); vitamin C (25.0 g); choline chloride (70.0 g); iodine (0.3 g); selenium (0.05 g); iron (6.0 g); copper (1.2 g); zinc (16.0 g); manganese (7.0 g); cobalt (0.1 g); and antioxidant (30.0 g)
